# Supplementary material for: Antioxidant Capacity, Vitamin C and Polyphenol Profile Evaluation of a Capsicum chinense By-Product Extract Obtained by Ultrasound Using Eutectic Solvent
Source: Plants (Basel). 2022 Aug 6;11(15):2060. doi: 10.3390/plants11152060 (PMC9370112; doi:10.3390/plants11152060)
Supplement: Supplementary file 1 [file plants-11-02060-s001.zip › plants-1841516-supplementary.pdf]

SUPPLEMENTARY MATERIAL

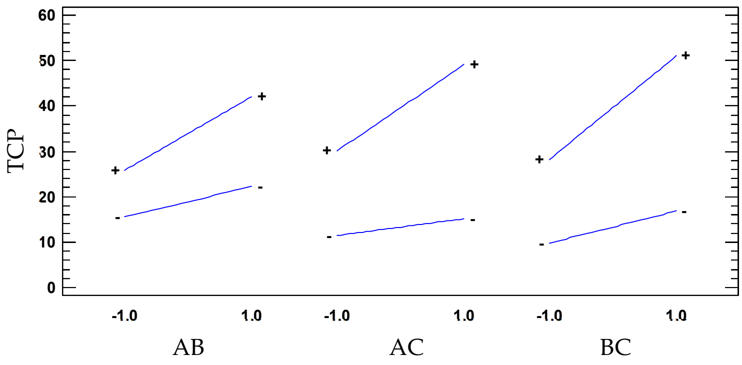

Figure S1. Interaction chart for total polyphenol content (TPC); Capital letters represent a main factor where A: Variety, B: By-product and C: solvent

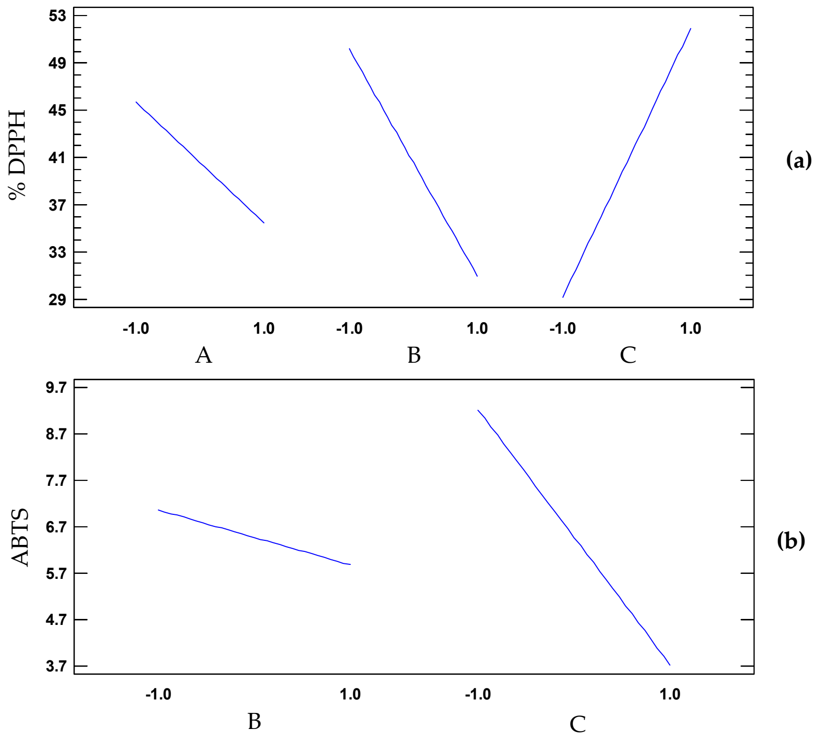

Figure S2. Main Effect plot; DPPH (a), ABTS (b). Capital letters represent a main factor where A: Variety, B: By-product and C: solvent

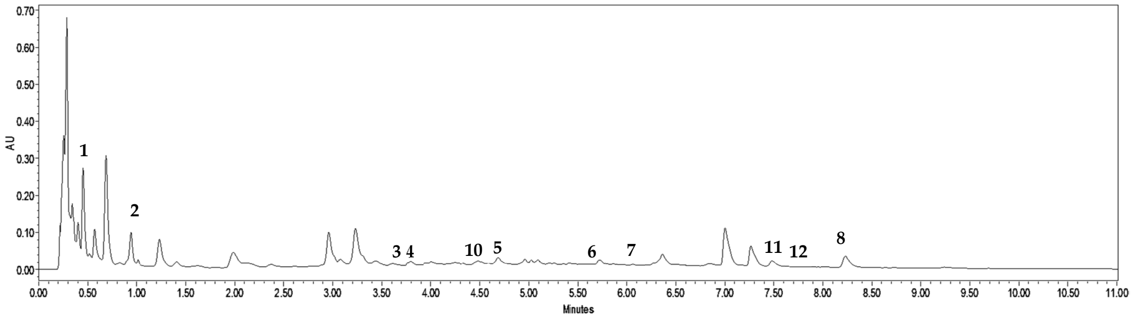

Figure S3. Chromatogram corresponding to Habanero pepper Jaguar leaf extract by NADES-h. Numeration: 1 = gallic acid; 2 = protocatechuic acid; 3 = catechin; 4 = chlorogenic acid; 5 = cumaric acid; 6 = cinnamic acid; 7 = rutin; 8 = quercetin + luteolin; 10 = vanillin; 11 = diosmin + hesperidin; 12= neohesperidin

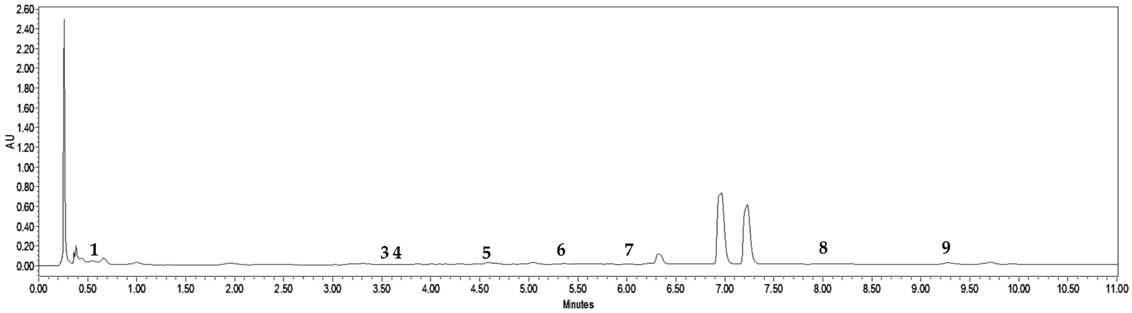

Figure S4. Chromatogram corresponding to Habanero pepper Jaguar leaf extract by MeOH. Numeration: 1 = gallic acid; 3 = catechin; 4 = chlorogenic acid; 5 = cumaric acid; 6 = cinnamic acid; 7 = rutin; 8 = quercetin + luteolin; 9 = kaempferol

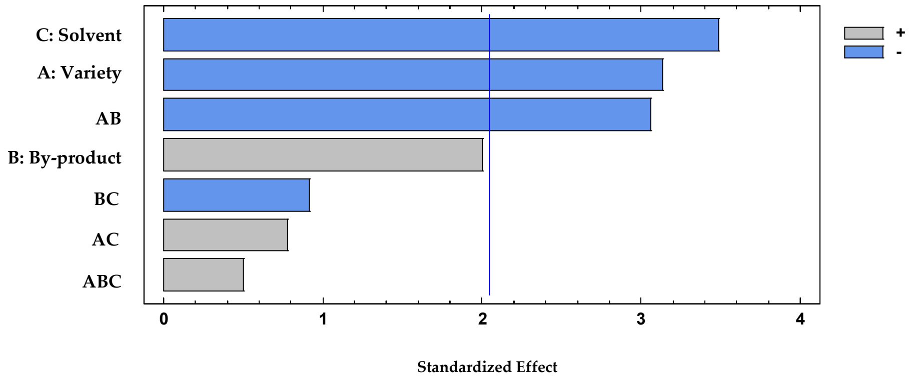

Figure S5. Pareto chart of vitamin C

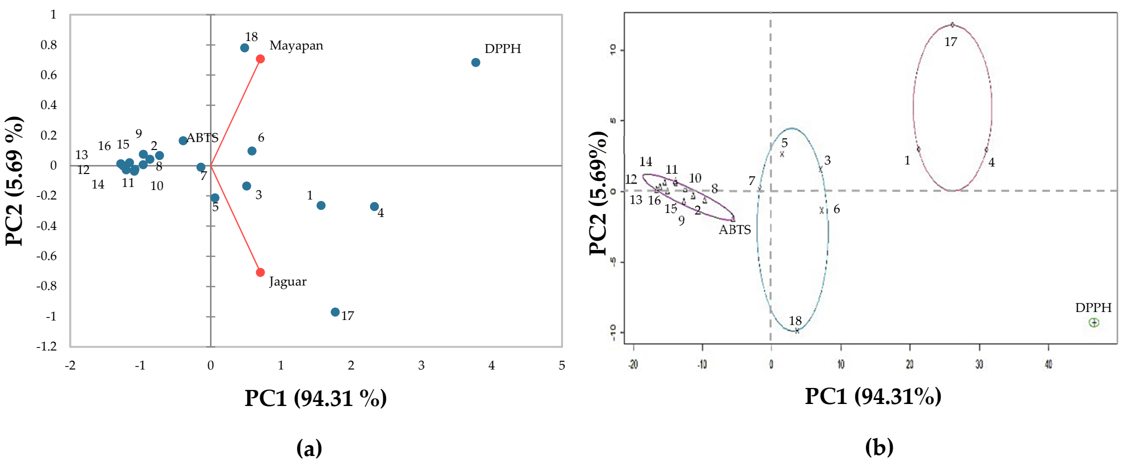

Figure S6. Analysis of total polyphenol content, polyphenol profile and antioxidant activity: Principal component analysis (PCA) depending on variety (a) and cluster of k means depending on variety (b) used. Numeration: 1 = Total polyphenol content; 2 = gallic acid; 3 = protocatechuic acid; 4 = catechin; 5 = chlorogenic acid; 6 = quercetin + luteolin; 7 = rutin; 8 = kaempferol; 9 = neohesperidin; 10 = diosmetin; 11 = vanillin; 12 = cumaric acid; 13 = cinnamic acid; 14 = naringenin; 15 = apigenin; 16 = lutein, 17 = diosmin + hesperidin; 18 = vitamin C

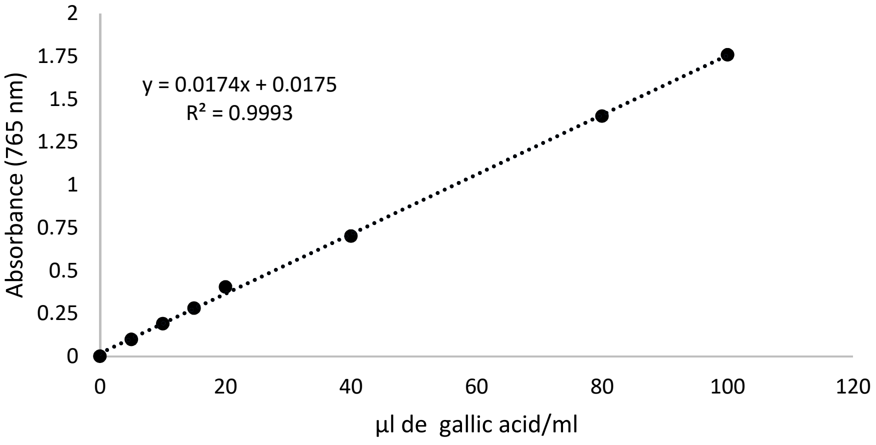

Figure S7. Total polyphenol content (TPC) calibration curve

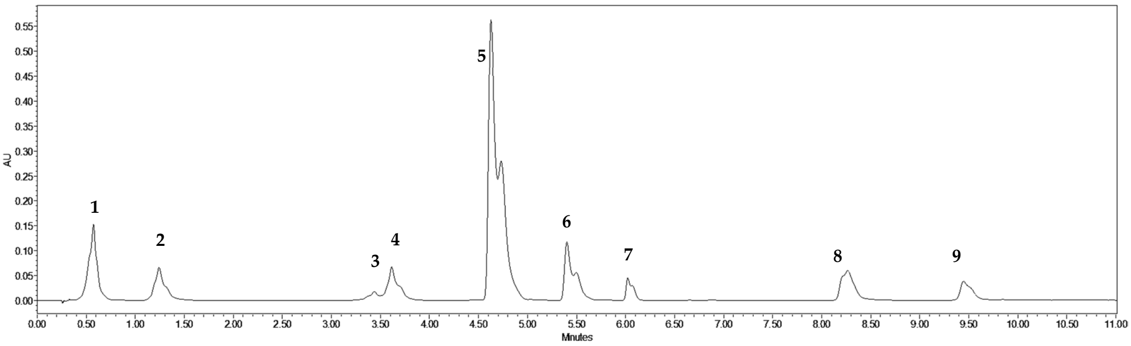

Figure S8. Chromatogram corresponding to the calibration curve with the polyphenol standards. Numeration: 1 = gallic acid; 2 = protocatechuic acid; 3 = catechin; 4 = chlorogenic acid; 5 = cumaric acid; 6 = cinnamic acid; 7 = rutin; 8 = quercetin + luteolin; 9 = kaempferol.

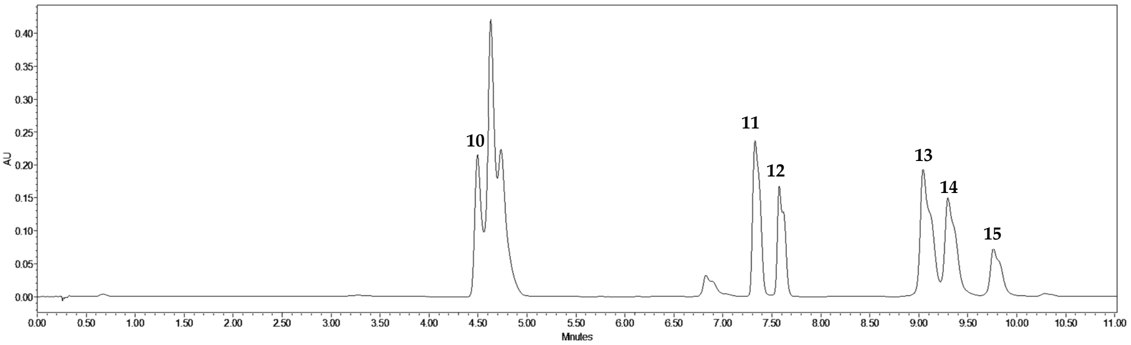

Figure S9. Chromatogram corresponding to the calibration curve with the polyphenol standards. Numeration: 10 = vanillin; 11 = diosmin + hesperidin; 12 = neohesperidin; 13 = naringenin; 14 = apigenin; 15 = diosmetin

Table S1. Statistical analysis of extracts polyphenol profile obtained from the habanero pepper (*Capsicum chinense* Jacq.) by-products

Factor	***p*-Value**							
	**Gallic acid**	**protocatechuic acid**	**catechin**	**chlorogenic acid**	**Coumaric acid**	**Cinnamic acid**	**rutin**	**quercetin + luteolin**
**A**	0.2244	0.3775	0.0260	0.0017	0.0000	0.0993	0.1879	0.6876
**B**	0.0274	0.4474	0.2242	0.0069	0.0000	0.0000	0.0000	0.0426
**C**	0.0327	0.5182	0.0001	0.0000	0.6986	0.3155	0.4618	0.6837
**AB**	0.2628	0.4590	0.9937	0.6921	0.0027	0.0180	0.5354	0.7846
**AC**	0.2388	0.6778	0.2017	0.0431	0.6268	0.9749	0.4566	0.9400
**BC**	0.6065	0.6708	0.0028	0.0403	0.6726	0.1151	0.3492	0.6229
**ABC**	0.2609	0.7334	0.5454	0.7557	0.6521	0.4684	0.9438	0.9443

Note. A = Variety; B = by-product; C = Solvent; n = 3

Tabla S1. (Cont.) Statistical analysis of extracts polyphenol profile obtained from the habanero pepper (*Capsicum chinense* Jacq.) by-products

Factor	*p*-Value						
	**kaempferol**	**vanillin**	**diosmin + Hesperidin**	**neohesperidin**	**naringenin**	**apigenin**	**diosmetin**
**A**	0.7014	0.0001	0.0184	0.7819	0.0299	0.6398	0.1480
**B**	0.0000	0.0000	0.0000	0.0141	0.0000	0.0006	0.0000
**C**	0.0000	0.0001	0.0007	0.9072	0.0025	0.0006	0.0022
**AB**	0.7649	0.0012	0.0226	0.3426	0.0299	0.6801	0.1480
**AC**	0.1842	0.5925	0.4618	0.5160	0.9221	0.8530	0.3313
**BC**	0.0000	0.7505	0.0006	0.7684	0.0025	0.0006	0.0022
**ABC**	0.1842	0.8772	0.5108	0.6363	0.9221	0.8530	0.3313

Note. A = Variety; B = by-product; C = Solvent; n = 3

Table S2. Equations derived from the linear regression of the individual polyphenols, vitamin C, lutein and the TPC with antioxidant activity

Polyphenols	DPPH		ABTS	
	**Stem**	**Leaf**	**Stem**	**Leaf**
Gallic acid	y = -0.062x + 7.6993	y = -0.1436x + 11.535	y = 0.1628x + 5.8997	y = 0.2936x + 4.7517
protocatechuic acid	y = -1.4699x + 65.044	y = -0.9756x + 64.042	y = 0.0732x + 5.7289	y = 0.0668x + 4.9298
catechin	y = -0.9807x + 71.773	y = -0.9422x + 82.141	y = 0.1009x + 4.0097	y = 0.0922x + 2.591
chlorogenic acid	y = -3.0633x + 73.06	y = -1.8447x + 74.221	y = 0.3361x + 3.6898	y = 0.2076x + 2.9314
Coumaric acid	y = -49.41x + 62.29	y = -18.333x + 67.811	y = 0.7691x + 6.434	y = 0.3484x + 5.8153
cinnamic acid	y = -11.982x + 76.048	y = -5.3354x + 62.704	y = 0.3481x + 5.8105	y = 0.0528x + 6.0765
rutin	y = -1.8388x + 62.19	y = -2.8839x + 79.06	y = 0.0289x + 6.4333	y = 0.1121x + 4.9194
quercetin + luteolin	y = -1.1964x + 64.377	y = -1.1317x + 65.323	y = 0.0394x + 6.0767	y = 0.0282x + 5.7412
kaempferol	y = -0.7339x + 48.332	y = 1.8068x + 37.5	y = -0.264x + 7.6403	y = -0.2903x + 7.411
vanillin	y = -27.18x + 77.945	y = -11.806x + 74.544	y = 2.0203x + 4.2952	y = 0.6833x + 4.527
diosmin + Hesperidin	y = 0.4098x + 38.916	y = 0.1832x + 37.156	y = -0.0618x + 7.7155	y = -0.0281x + 7.4125
neohesperidin	y = -6.6742x + 66.416	y = -2.5337x + 51.499	y = 0.2825x + 5.8153	y = -0.0643x + 6.4271
naringenin	y = 32.289x + 39.173	y = -7.4156x + 48.649	y = -4.0325x + 7.507	y = -0.9025x + 6.7355
apigenin	y = -2.9205x + 48.855	y = 5.208x + 38.237	y = -0.8412x + 7.6021	y = -0.9373x + 7.4174
diosmetin	y = 4.8037x + 39.173	y = 2.7993x + 37.719	y = -0.5999x + 7.507	y = -0.3892x + 7.2249
TPC	y = 0.42x + 36.969	y = 0.5577x + 26.781	y = -0.1718x + 10.262	y = -0.127x + 10.334
Vitamin C	y = -0.1165x + 17.851	y = -0.3074x + 30.794	y = 1.8666x - 1.1828	y = 3.2987x - 0.9657

Note: TPC = Total polyphenol content
